# E-ACTIVE AGING study protocol: Evaluating an exergame-based and multicomponent exercise program for community-dwelling older adults at risk of falling

**DOI:** 10.3389/fphys.2025.1691454

**Published:** 2025-12-03

**Authors:** Paloma Lillo-Urzúa, Jorge Ugarte-Llanten, Gabriel Carreño-Zilmann, Nicolás Vidal-Seguel, Francisco Guede-Rojas, Magdalena Cuenca-García, Igor Cigarroa

**Affiliations:** 1 Escuela de Kinesiología, Facultad de Ciencias de la Salud, Universidad Católica Silva Henríquez, Santiago, Chile; 2 Center for Social and Cognitive Neuroscience, School of Psychology, Universidad Adolfo Ibáñez, Santiago, Chile; 3 Universidad de La Frontera, Facultad de Medicina, Departamento de Ciencias Básicas, Temuco, Chile; 4 Universidad de La Frontera, Facultad de Medicina, Doctorado en Ciencias Morfológicas, Temuco, Chile; 5 Exercise and Rehabilitation Sciences Institute, School of Physical Therapy, Faculty of Rehabilitation Sciences, Universidad Andres Bello, Santiago, Chile; 6 GALENO Research Group, Department of Physical Education, Faculty of Education Sciences, School of Education, University of Cádiz, Cádiz, Spain; 7 Instituto de Investigación e Innovación Biomédica de Cádiz (INiBICA), Cadiz, Spain; 8 Facultad de Ciencias de la Salud, Universidad Arturo Prat, Victoria, Chile

**Keywords:** exergame, risk of falls, older adults, static and dynamic balance, strength, functional capacity, multicomponent exercise

## Abstract

**Background:**

Falls among older adults represent a major cause of morbidity and mortality, leading to decreased physical activity, loss of independence, and increased dependency. Individuals aged 60 years and older, particularly those with sensory deficits, are at greater risk. While conventional fall-prevention programs are widely implemented, innovative strategies such as active exergames have emerged as promising approaches to enhance balance and reduce fall risk.

**Objective:**

This study aims to evaluate the effectiveness of a supervised exergame-based multicomponent intervention compared to a traditional multicomponent training program in community-dwelling older adults at risk of falling.

**Methods:**

A randomized controlled trial with parallel groups and blinded assessment will be conducted among older adults (≥60 years) recruited from senior centers (SENAMA, Chile). Fifty-two participants will be randomly allocated to either an exergame group (n = 26), performing interactive full-body movements using the Nintendo Switch^®^, or a traditional multicomponent training group (n = 26). Both groups will receive 1-h supervised sessions twice weekly for 12 weeks. The primary outcomes will include fall risk, balance performance, functional independence and cardiorespiratory fitness. Secondary outcomes will assess body composition, muscular strength and quality, physical activity level, quality of life, and pain intensity and interference.

**Expected Results:**

It is hypothesized that both interventions will improve functional and balance outcomes, with the exergame-based program potentially promoting greater adherence and superior overall effects.

**Clinical Trial Registration:**

ClinicalTrials.gov. Identifier: NCT07024004.

## 1 Introduction

The global population is aging rapidly, and the number of people aged 65 years and older is projected to more than double between 2020 and 2050, reaching approximately 16% of the world’s population (Rubenstein, 2006). Falls represent one of the leading causes of morbidity and mortality in this demographic, often resulting in mobility limitations, reduced quality of life, and loss of Independence (Rubenstein, 2006). Declines in lower-limb strength, impaired balance, and decreased functional mobility are key modifiable risk factors for falls (Pijnappels et al., 2008; Pijnap et al., 2008; Hamacher et al., 2018; Guideline for the prevention of falls in older persons. American Geriatrics Society, 2001). Targeted exercise interventions can mitigate these physiological declines by improving neuromuscular performance and thereby reducing the risk and incidence of falls (Shumway-Cook et al., 2007; Sherrington et al., 2011). Evidence supports the benefits of multicomponent exercise programs—including strength, balance, flexibility, and aerobic training—in maintaining functional capacity, preventing chronic diseases, and enhancing quality of life among older adults (Schneider et al., 2025; Li et al., 2025; Casas-Herrero et al., 2022). Such programs are most effective when they are personalized, supervised, and engaging, as these factors promote adherence and sustained participation (Reyna et al., 2020).

In recent years, technology-based exercise interventions have gained attention as innovative tools to increase motivation and long-term adherence among older adults. Exergames—interactive video games that combine exercise with virtual environments—offer a safe and enjoyable way to train balance, coordination, and strength (Valenzuela et al., 2018; Lee, 2013; Fang et al., 2020; Street et al., 2017). Research has shown that exergames can improve postural control, muscle strength, and functional independence in older adults and individuals with neurological conditions, while also enhancing psychological wellbeing and enjoyment compared to traditional exercise (Schroeder, 2008; Lavoie et al., 2025; Oh and Yang, 2010; Mejia-Downs et al., 2018; Bieryla and Dold, 2013; Rendon et al., 2012; O’Loughlin et al., 2020). Commercial platforms such as the Nintendo Wii®, Xbox Kinect®, and, more recently, the Ring Fit Adventure for Nintendo Switch® provide accessible options for such interventions (Li et al., 2018; Chua et al., 2013; Tanaka et al., 2012).

Despite these promising results, most studies to date have focused on short-term or unsupervised exergame interventions and have used heterogeneous technologies, making it difficult to determine which specific modalities or implementation strategies are most effective for fall prevention in older adults (Efficacy of Nintendo, 2018; Orsega-Smith et al., 2021). Furthermore, limited evidence exists comparing exergame-based programs integrated with multicomponent exercise against traditional exercise protocols under supervised conditions.

Therefore, this study aims to address this knowledge gap by evaluating the effectiveness of a supervised exergame-based multicomponent intervention compared with a traditional multicomponent training program in older adults at risk of falling. By combining evidence-based exercise principles with interactive digital technology, this study seeks to determine whether such integration can enhance adherence and yield superior improvements in balance, functional performance, and overall wellbeing.

## 2 Aim

This study aims to evaluate the effectiveness of a supervised exergame-based multicomponent intervention on balance, cardiorespiratory fitness, muscle strength, and body composition in older adults at risk of falling. By providing context-specific evidence, this research seeks to determine whether integrating exergames into conventional training programs can enhance fall-prevention strategies and improve quality of life in this population.

## 3 Methods

### 3.1 Study design

This study is designed as a blinded (assessor and statistician), parallel group, randomized controlled trial (RCT). CONSORT guidelines for clinical trials will be followed (Hopewell et al., 2025), reporting on social and psychological interventions (Montgomery et al., 2018), and the SPIRIT guidelines for protocol studies (Chan et al., 2013) (Supplementary Table 1). The following ethical implications are considered: the signature of the informed consent of each subject before participation; voluntary participation; data will be handled anonymously; the private data of the participants will be anonymized, and we adhere to the international regulations on human research following the principles established in the Declaration of Helsinki (Serra, 2025). The protocol was submitted and approved to the ethical-scientific committee of Universidad Santo Tomás (Chile) (code n°238-MZS, Approval date 24 November 2024). In addition, the clinical trial was registered on ClinicalTrials.gov (ID: NCT07024004).

### 3.2 Study setting and participants

National service for the elderly (SENAMA, in Spanish) in the Puente Alto district, metropolitan region, Santiago, Chile, will be invited to participate. SENAMA day center offers social and health services to the elderly during the day, with the aim of promoting their autonomy and independence, and preventing loss of functionality. These centers provide support through workshops and an individualized intervention plan, seeking to keep the elderly in their family and social environment. This day center was selected because it is the center that receives the largest number of elderly people in the metropolitan region. This provides access to a diverse group of older adults, including those who are frail and meet the study’s selection criteria. A collaboration agreement was created with SENAMA to be able to carry out outcome measurements and both protocols of exercise. In addition, this day center has a space equipped with a 50-inch smart TV and physical activity equipment so that a health professional can guide all the sessions.

### 3.3 Power calculation

**FIGURE 1:**
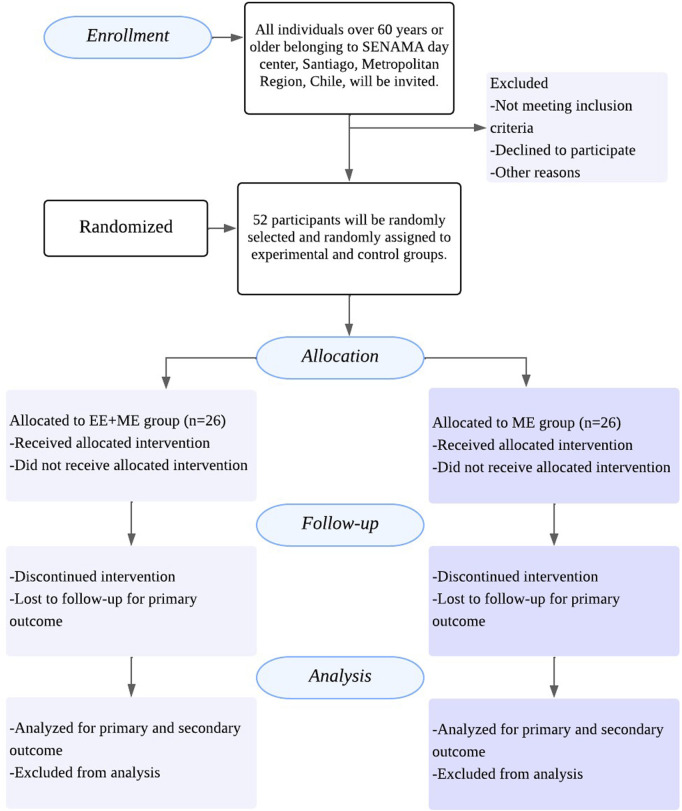
Flowchart of active exergame intervention (Hopewell et al., 2025). EE + ME Group: Exergame-based training plus multicomponent exercise group; ME Group: Multicomponent exercise group.

The sample size was calculated as *a priori* using G*Power 3.1.9.7. Forty-six participants in total are required (23 in each group), considering α = 0.05, 1-β = 0.8, and effect size (ES) = 0.8 (Lower limb strength based on the results of a previous study) Considering the possibility of dropouts percentage, six participants were added (see Figure 1).

### 3.4 Recruitment of participants

All individuals belonging to SENAMA day center will be invited to participate. Research support staff will conduct recruitment in person. To be eligible for participation, individuals must meet the following inclusion criteria: (i) both sexes eligible; (ii) aged ≥60 years; (iii) attendees of SENAMA day centers (senior care centers) in the Metropolitan Region of Santiago, Chile; (iv) Short Mini-Mental State Examination (MMSE-S) score >14 (Jiménez et al., 2017); (v) Functional independence measure (FIM), Index score >60 (indicating moderate to good functional independence) (Paolinelli et al., 2001); (vi) no self-reported medical contraindications for physical exercise and; (v) ability to walk independently (with or without cane). Exclusion criteria: (i) recent bone fractures or acute myocardial infarction; (ii) severe cardiovascular or respiratory conditions; (iii) uncontrolled hypertension or diabetes mellitus; (iv) cognitive or sensory impairments that limit instruction comprehension; (v) participation in other exercise programs during the study period and; (vi) inability to attend at least 80% of the intervention sessions (see Figure 1).

### 3.5 Randomization

The sample was selected from among those attending the SENAMA day center, reaching a size of 52 participants. The selection was randomized among those who met the inclusion and exclusion criteria. Participants were selected according to predefined inclusion and exclusion criteria. Inclusion criteria comprised adults aged 60 years or older of both sexes, attending SENAMA day centers in the Metropolitan Region of Santiago, Chile, with a Mini-Mental State Examination (MMSE) score greater than 14 and a Barthel Index score above 60, indicating moderate to good functional independence. Participants were required to walk independently (with or without assistive devices), report no medical contraindications for physical exercise, and provide written informed consent. Exclusion criteria included recent bone fractures or acute myocardial infarction, severe cardiovascular or respiratory conditions, uncontrolled hypertension or diabetes mellitus, cognitive or sensory impairments limiting instruction comprehension, concurrent participation in other exercise programs, or inability to attend at least 80% of intervention sessions. Participants were randomized by research staff using a computer-generated sequence and concealed allocation to one of two groups (Rubenstein, 2006): the exergame-based training plus multicomponent exercise group (EE + ME group), which will receive an exergame-based program combined with multicomponent exercise, or (Pijnappels et al., 2008) the multicomponent exercise group alone (ME group), which will receive multicomponent exercise. A computer-generated simple randomization sequence stratified by gender, age, and falls risk was used to balance the groups by sex, age, ranges, and falls risk index (RRI) in both groups. The randomization sequence was concealed using sealed, opaque, sequentially numbered envelopes to ensure allocation concealment (see Figure 1).

### 3.6 Procedure

**FIGURE 2:**
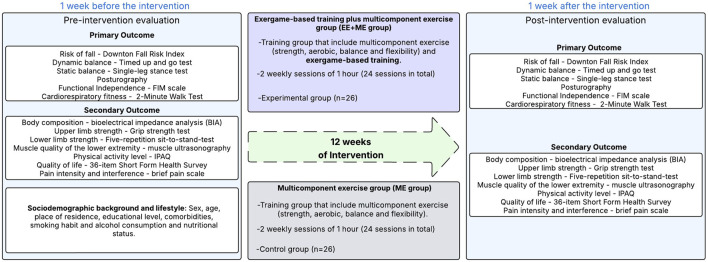
Study design showing pre-and post-intervention assessments and allocation of participants to two groups (multicomponent exercise with or without exergame-based training) during a 12-week protocol.

Primary outcomes (risk of falls, functional independence, dynamic and static balance, center of pressure (CoP) and cardiorespiratory fitness) and secondary outcomes (body composition, upper limb strength, lower limb strength, lower limb quality muscle, quality of life and pain intensity and interference) will be assessed face-to-face 2 weeks prior to the initiation of the intervention in both groups (see Figure 2). One week prior to the intervention, there will be two induction sessions on the proper use of sports equipment and exergame use.

At the beginning of the interventions, all participants will receive an adverse symptom briefing and will be trained with the Rating of Perceived Exertion (RPE) with modified Borg Scale (Borg, 1982) to report their perception before, during and at the end of the sessions.

Throughout the 12-week intervention period, participants will be encouraged to maintain the same levels of physical activity and caloric intake as before participating in the study. Participants should report any changes in physical activity levels or nutritional intake to the physiotherapist delivering the exercise sessions. The physiotherapist will record their observations on the attendance record.

A post-intervention assessment will be conducted after the last week of the 12-week program (see Figure 2). All data collection will be carried out in a face-to-face meeting with participants at SENAMA day center and in the physiology laboratory of the Silva Henríquez Catholic University. Technical staff will be trained to ensure an unbiased procedure. Measurements will be taken individually in a place with optimal conditions of privacy, temperature, and humidity. Evaluations will be conducted by healthcare professionals. The person evaluating will be different from the person performing the interventions.

### 3.7 Interventions

**FIGURE 3:**
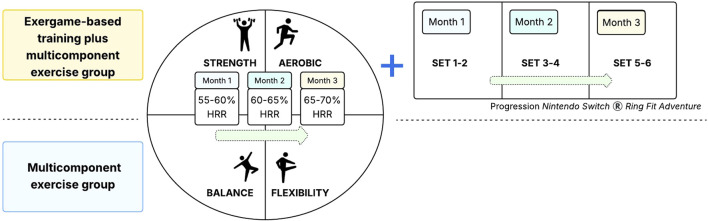
Schematic representation of the progression in the multicomponent exercise program based on heart rate reserve (HRR), and the incremental intensity of exergame training using Nintendo Switch® Ring Fit Adventure, with more complex sets introduced as the intervention months advance.

Exercise sessions will be held at the SENAMA day center. Regardless of the group (multicomponent exercise or multicomponent exercise plus active exergames), each session will consist of a warm-up activity (5–10 min), a main exercise activity (50 min) and a cool-down activity (5–10 min) following the guidelines of the Vivifrail multicomponent exercise program (Norton et al., 2010). Exercise intensity will be monitored based on RPE on the Borg scale modified (Borg, 1982) and vital sign parameters such as blood pressure, heart rate and saturation will be assessed before, during and after each exercise session according to the American College of Sports Medicine (ACSM) guidelines (ACSM’s, 2010), as explained below. Warm-up and cool-down activities will include stretching and walking in place (4≤RPE≤5 and 40 < 55% of heart rate reserve (HRR), while the main exercise activity will consist of strength, endurance, and balance (falls avoidance) exercises performed with dumbbells, color-coded resistance bands (Thera-Band*®*; Hygienic Corp.) Ferretti et al., 2020, and a chair (4 < RPE ≤ 6 and 55 < 70% HRR) (see Figure 3).

To ensure safety and compliance, physical training will be supervised by a healthcare professional (physiotherapist) previously trained in the use of exergames. The physiotherapist will provide personalized instructions to each participant based on the modified Borg scale target and HRR depending on exercise progression. Each group will be assigned to physiotherapist with the corresponding protocol. The exergames-based training group plus multicomponent exercise will have a 55-inch television to ensure that Exergames can be viewed. During each game, participants will be required to carefully follow the instructions of a virtual trainer and control an avatar while simultaneously receiving visual, auditory, and tactile feedback. The physiotherapist will have a safe exercise protocol (for adverse events and side effects of exercise). A detailed report of the elements of the intervention is delivered through the TiDIER checklist (template for intervention description and replication) (Supplementary Table 2) (Hoffmann et al., 2014).

#### 3.7.1 Exergame-based training plus multicomponent exercise group (EE + ME group)

**FIGURE 4:**
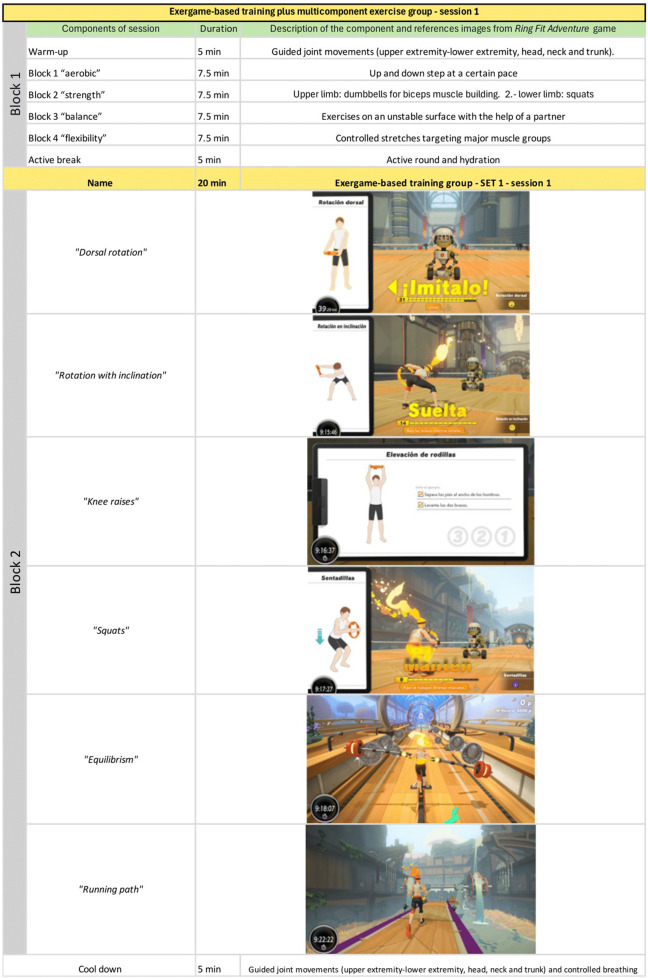
Example of structure and components of Session 1 for EE-ME group.

The video game-based training intervention will be carried out individually. The session will have a warm-up activity (5 min), main exercise activity divided into block 1 (ME) and 2 (EE) will last for 50 min, and cool-down activity (10 min), following the guidelines of a multicomponent exercise program and defined exergame use protocol. Exercise intensity will be monitored on the modified Borg scale. Warm-up and cool-down activities will include stretching, joint mobility and walking in place (4 < RPE ≤ 5 and 40 < 55% of HRR), while the main activity, in block 1, will consist of four exercise sub-blocks of 7.5 min each (strength, aerobic, balance and flexibility sub-blocks) performed with dumbbells, color-coded resistance bands (Thera-Band; Hygenic Corp.) and a chair (6<RPE≤7 and 55 < 60% of HRR in the first month and with its subsequent progression) (see Figure 3). Following the multicomponent exercise, a 5-min break will be given for hydration and continue with block 2 with Exergames (see Figure 4).

The exergame-based training session will last 20 min thus ensuring an equivalent exercise time in both groups (50 min per session) where participants will perform the set defined by the physiotherapist, for each week and month, according to the progression (see Figure 3). They included a series of analytical exercises, yoga postures, and playful physical activities from the game Ring Fit Adventure (Nintendo Switch®). The exergame protocol has a variety of sets (see Table 1), which will be adapted according to the progression of the exercise. The list of individual exergame activities and their description is provided in Supplementary Tables 3a b *, respectively* (Guede-Rojas et al., 2025). Finally, the session will culminate with a cool down (10 min).

**TABLE 1:** Risk of fall and functional capacity, physiological, and self-reported outcomes.

Domain	Outcome	Measurement tool/Test	Units/Scale
Risk of fall and functional capacity	Fall risk	Downton Fall Risk Index (DFRI)	0–11 points (≥3 = high risk)
	Dynamic balance	Timed Up and Go (TUG) test	Time (s), best of 3 trials
	Static balance	Single-Leg Stance Test	Time (s), best of 3 trials
		Posturography (Smart Balance Hur® Labs BTG4-F)	Romberg quotient; C90 area (cm²)
	Cardiorespiratory fitness	2-Minute Walk Test	Number of steps in 2 min
Physiological	Upper-limb strength	Handgrip Strength Test (BASELINE® dynamometer)	Force (kg), best of 2 trials
	Lower-limb strength	Five-Repetition Sit-to-Stand Test	Time (s)
	Muscle quality	Ultrasound (quadriceps, tibialis anterior) analyzed with Image J®	Muscle thickness (mm) and echogenicity (0–255 a.u.).
	Body composition	Bioelectrical Impedance Analysis (InBody 270®)	Skeletal mass, segmental muscle mass and fat mass (kg and %).
Self-reported	Functional independence	Functional Independence Measure (FIM)	18 items, 7-point scale (1 = dependence – 7 = independence)
	Quality of life	36-Item Short Form Health Survey (SF-36, Spanish version)	0–100 points (higher = better)
	Physical activity	International Physical Activity Questionnaire (IPAQ)	MET-min per week
	Pain intensity & interference	Brief Pain Inventory – Short Form (BPI-SF)	0–10 numeric scales (average score)

#### 3.7.2 Multicomponent exercise group (ME group)

The intervention will be conducted in a group. Each session will have a warm-up activity (5 min), main exercise activity (50 min), following the guidelines of a multicomponent exercise program. Exercise intensity will be monitored according to the modified Borg scale. Warm-up and cool-down activities will include stretching, joint mobility and walking in place (four to five RPE and 40 < 55% of HRR), while the main activity will consist of four exercise blocks of 12.5 min each (strength, aerobic, balance and flexibility sub-block) performed with dumbbells, color-coded resistance bands (Thera-Band®; Hygenic Corp.) and a chair (6 < RPE ≤ 7 and 55 < 60% RHR in the first month and with its subsequent progression) (see Figure 3). Finally, the session will culminate with a cool down (10 min).

### 3.8 Outcome measures

Physical fitness, functional status and quality of life will be assessed before and after the interventions. All measurements will be performed by two physiotherapists who are not familiar with the group assignment scheme, which will ensure an unbiased procedure. In addition, all measurements will be performed in the physiology laboratory of Católica Silva Henríquez University and will be obtained individually in an environment with optimal conditions of privacy, temperature and humidity. Data will be recorded on standardized forms and entered into a secure access database containing quality control checks (e.g., range checks and missing data notifications). The primary outcomes were selected based on studies that analyzed core outcome sets recommended for evaluating the effectiveness of interventions in people at risk of falls, as reported in the COMET initiative. This approach ensures that the selected outcomes are clinically meaningful, internationally comparable, and aligned with best practice standards (O’Malley et al., 2023; Lamb et al., 2005; Copsey et al., 2016) Thus, will include risk of fall, functional independence, measures of static and dynamic balance, center of pressure, cardiorespiratory fitness; while secondary outcomes will assess body composition, upper and lower limb strength, muscle quality of the lower limb, physical activity level, perceived quality of life and perceived pain. The variables are summarized according to three main domains and are presented in Table 1.

#### 3.8.1 Primary outcomes

Risk of fall: Falls risk will be evaluated using the Downton Fall Risk Index (DFRI), a five-item composite score based on prior falls, medication, sensory deficits, mental state, and mobility. A score of less than three points means low risk of fall, and a total score above three points indicates a high risk of fall (Downton index, 2016).

Balance: Dynamic balance will be measured with the Timed Up and Go (TUG) test, which evaluates the time in seconds that a participant takes to rise from a chair, walk 3 m, turn, return, and sit down again (Mancilla et al., 2015) The test will be assessed three times, and the best attempt will be recorded. While static balance will be measured thought the single-Leg Stance Test, where participants must maintain balance on one leg for at least 5 s (Mancilla et al., 2015) The test will be assessed three times, and the best attempt will be recorded.

Posturography: Romberg quotient and C90 area will be measured (Smart Balance Hur® Labs model BTG4-F, Kokkola, Finland). Posturography records center of pressure displacement while standing on a stable platform (Furman, 1994; Visser et al., 2008). Participants must maintain a bipedal posture with feet shoulder width apart, hands hanging at the sides of the body and head facing forward. They must also follow the evaluator’s instruction, either eyes open (condition 1) or closed (condition 2). The foot resting points on the platform are marked to ensure repeatability.

Functional independence: this outcome will be measured with Functional Independence Measure (FIM) scale, it consists of 18 items that assess both motor and cognitive skills, and is scored on a 7-level scale, where one indicates total dependence and seven complete independence (Paolinelli et al., 2001).

Cardiorespiratory fitness: Aerobic capacity will be measured by the 2-Minute Walk Test, which measures the total distance (in meters) covered at a self-selected walking pace within 2 min on a flat surface, a full step is defined as a step performed by raising the knee to a height corresponding to the midpoint between the patella and the iliac crest (Guralnik et al., 1994).

#### 3.8.2 Secondary outcomes

Body composition: Total body mass, fat mass, fat-free mass, visceral fat and segmental muscle mass of the lower limbs will be assessed using a bioelectrical impedance analyzer (InBody 270®, Biospace, Seoul, Korea) according to the manufacturer’s instructions (Sergi et al., 2015).

Upper limb strength: This will be estimated using the grip strength test, which uses a previously calibrated BASELINE® hydraulic dynamometer. The participant will sit on a chair with a backrest, with their shoulders adducted, their elbow flexed at 90°, and their forearm and wrist in a neutral position. The evaluated arm should not rest on any surface, and the dynamometer should be used in a vertical position. Participants will be asked to perform a maximum grip force with their dominant hand for 3 s, resting for 1 min between each repetition, making two attempts. The highest grip value of the repetitions will be used. This protocol has been described previously (EPI–Departamento de Epidemiologia, 2015).

Lower limbs strength: This will be measured using a five-repetition sit-to-stand test. Participants will be asked to stand up and sit down, as quickly as possible, five times from a chair without armrests located on a wall. The time will be measured from the beginning of the movement until participants manage to stand up for the fifth repetition. Arms should be crossed over the chest during the test. The time will be recorded in seconds and tenths of a second (Guralnik et al., 1994; Bohannon, 2006).

Muscle quality of the lower limb: Muscle quality will be evaluated through intramuscular fat infiltration, in which muscle echogenicity will be measured by ultrasound (Cross-sectional view of the quadriceps and tibialis anterior muscles). The images will be analyzed using ImageJ® software to calculate the gray scale intensity (0-255 arbitrary units), which reflects the intramuscular fat content and in addition the muscle architecture will be measured through muscle thickness (Lizama-Pérez et al., 2023).

Physical activity level: The level of physical activity in older adults can be measured using the IPAQ questionnaire, which has been shown to be a valid and reliable tool in older adults (Rubio Castañeda et al., 2017). Recent studies show that, although there is a tendency to overestimate physical activity, the IPAQ remains useful for population studies (Cleland et al., 2018) and has been used successfully in adults over 70 years of age, differentiating activity levels according to age and comorbidities (Ferretti et al., 2020).

Quality of life: This will use the 36-item Short Form Health Survey (SF-36) questionnaire (Vilagut et al., 2005) in its Spanish version (Alonso et al., 1995). The SF-36 is a self-report instrument that contains 36 questions from eight dimensions related to people’s health: physical function, physical role, bodily pain, vitality, social function, emotional role, mental health, and general health (Ware and Sherbourne, 1992). The score obtained corresponds to values on a scale of 0–100, where higher scores indicate better health (Ware and Sherbourne, 1992). The SF-36 is adapted and validated in Chilean older adults, presenting adequate indicators of general Cronbach’s alpha internal consistency of 0.88 (Lera et al., 2013).

Pain intensity and interference: The Brief Pain Inventory-Short Form (BPI-SF) remains one of the most validated and widely used tools for assessing pain intensity and interference across various clinical populations, especially in musculoskeletal and chronic pain conditions. Recent studies have confirmed its strong psychometric properties, including internal consistency (Cronbach’s α = 0.83–0.96), known-group validity, and responsiveness to change (U Jumbo et al., 2020). In a systematic review, the BPI-SF demonstrated a stable two-factor structure and was shown to be more robust and reliable than comparable tools such as the Short-Form McGill Pain Questionnaire-2 in musculoskeletal pain assessment (MacDermid et al., 2019). Additionally, the BPI-SF has been successfully adapted for older adult populations, including those with nociceptive, neuropathic, and nociplastic pain, with excellent test-retest reliability (ICC = 0.90–0.96) and internal consistency (α = 0.87) (Ferreira et al., 2023).

#### 3.8.3 Sociodemographic background

The sociodemographic characteristics of the older adults will be recorded, including age, sex (male/female), geographic origin (rural or urban), educational level (basic education: <8 years, secondary education: 8–12 years, higher education: >12 years), and the presence of comorbidities such as diabetes mellitus, hypertension, and dyslipidemia, as well as some habits such as smoking habit and alcohol consumption. Nutritional status will be classified according to body mass index (BMI), which is obtained by dividing body weight by bipedal height squared (weight/height^2^), with cut-off points being considered, underweight <22.9 kg/m^2^, normal weight: 23.0–27.9 kg/m^2^, overweight: 28.0–31.9 kg/m^2^, and obese: ≥32.0 kg/m^2^ Reis et al., 2019. The questions and classification indicated in the National Health Survey 2016-2017 will be used (EPI–Departamento de Epidemiologia, 2015).

### 3.9 Data management, data access and dissemination policy

Data collection will be carried out in a SENAMA center located in Santiago, Chile, and will be in charge of a team of four researchers, who will apply the instruments contemplated in the intervention protocol. All collected data will be anonymized at the point of entry, ensuring that no personal identifiers are stored in the database. The anonymized dataset will be stored on the institutional Dropbox® platform, which is protected by SSL/TLS encrypted transfer, AES 256-bit encryption at rest, and two-factor authentication. Access to the repository will be strictly limited to the principal investigator and co-researchers through secure institutional accounts. Data will be stored for a period of 5 years following the completion of the study, in accordance with the data protection policies of Universidad Católica Silva Henríquez and national ethical regulations in Chile (Law 20.584 on patient rights and duties). After this period, all data will be permanently deleted. The use of Dropbox® has been reviewed and approved as part of the institutional data management policy, ensuring compliance with ethical and confidentiality standards.

Once the study is completed, the results obtained will be shared with SENAMA, as well as with the academic community through presentations at specialized national and international congresses and seminars. In addition, the findings, including analyses derived from mechanical inference, will be published in scientific journals in the field, with the aim of contributing to the body of knowledge on innovative intervention strategies for older adults.

### 3.10 Statistical analysis

Missing data will be handled by intention-to-treat analysis (multiple imputation method) [65]. The data will be analyzed using the intention-to-treat (ITT) strategy (Ahn and Kang, 2023). The description will be made as measures of central tendency and dispersion (continuous variables) and as percentages (categorical variables). Using the Shapiro-Wilk test, the normality of the data will be verified, and a two-step approach for transforming will be applied to the non-normal variables (A Two-Step Approach for Transforming, 2004). Homoscedasticity will be analyzed using Levene’s test. A 2-way repeated measures analysis of variance (ANOVA) will be used to determine the effects of interventions. The effects of the model are the group (GE; GC), the times (Pretest; Posttest), and their interaction over time (Time x Group). The effect size (ES) will be determined using Cohen’s d (<0.2 insignificant; ≥0.2 and ≤0.49 small; ≥0.5 and ≤0.79 moderate; ≥0.8 large) (Cohen, 2004). Additionally, in clinically relevant outcomes (risk of fall), the minimum clinically important difference (MCID) will be evaluated. All analyses will be performed with SPSS® v.26 (SPSS, Inc., Chicago, IL, USA), considering p < 0.05 and GraphPad Prism® v8.

## 4 Discussion

The primary objective of this study is to evaluate the effectiveness of a supervised exergame-based plus traditional multicomponent intervention compared to a traditional multicomponent training program in older adults at risk of falling. Based on the literature, it is hypothesized that both interventions will yield positive effects on primary and secondary outcomes. However, it is expected that the exergame-based program may result in equal or superior outcomes compared to the traditional intervention. This expectation is based on evidence suggesting that exergames can enhance exercise adherence and promote sustained engagement, which are often barriers in older adults (Valenzuela et al., 2018). Exergames have been described as an effective strategy to improve adherence to physical activity, particularly when integrated with social interaction and individualized support. Previous systematic reviews and meta-analyses have shown that exercise-based interventions benefit multiple domains of health in older adults, including functional capacity, balance, cardiorespiratory fitness, muscle strength, and quality of life, while also reducing fall risk (Fang et al., 2020; MacDermid et al., 2019; Ferreira et al., 2023; Ahn and Kang, 2023; A Two-Step Approach for Transforming, 2004; Cohen, 2004). These outcomes align with the anticipated benefits of both interventions in this study. However, while exergames show promise, there is limited evidence regarding their long-term impact on physical activity levels and fall prevention in older adults (Reis et al., 2019; Schoene et al., 2013). Additionally, exergames require specific equipment, and the cost of this technology remains a potential barrier for widespread implementation in day centers or community-based settings (Kari, 2014; Digitally, 2016).

While the potential benefits of incorporating exergames in elderly care settings are clear, challenges such as motivation to engage in physical activity, the perception of reduced social support when attending non-group-based interventions, and the financial cost of technological devices must be considered (Schürch et al., 2023). Despite these challenges, the personalized nature of the exergame-based program in this study, with direct supervision from trained professionals, is expected to mitigate some of these limitations by ensuring adherence to exercise guidelines and encouraging consistent engagement.

### 4.1 Strengths, limitations, applications, and future research

A key strength of this study lies in its relevance and timeliness as a study protocol focused on fall prevention and functional improvement in older adults through exergame-based and multicomponent exercise interventions. The topic is highly pertinent given the growing need for innovative, technology-supported physical activity strategies addressing the challenges of ageing populations. Nevertheless, a potential limitation concerns the difficulty of achieving adequate exercise adherence among older adults, particularly when compared to established programs such as Vivifrail®. In terms of applications, this project underscores the importance of implementing such interventions worldwide, as aging and falls represent a significant and ongoing global public health concern. Future research should explore the development of exercise protocols incorporating other types of virtual reality environments, as well as the creation of accessible digital platforms for older adults who are unable to attend in-person sessions at community or care centers.

## 5 Conclusion

The combined intervention of exergame-based training and multicomponent exercise is expected to improve functional, cognitive, and exercise adherence outcomes in older adults. While multicomponent exercise alone can enhance functional capacity, balance, muscle strength, body composition, quality of life, and pain intensity, integrating active technologies such as exergames may further amplify these benefits by increasing motivation and engagement. This approach holds translational potential for implementation in day centers and senior living communities, supporting the promotion of physical activity through accessible and enjoyable tools.

Although challenges such as equipment costs and maintaining social interaction remain, a personalized and professionally supervised design may help mitigate these barriers. Future research should explore long-term effects, cost-effectiveness, and scalable models of integration into community and healthcare settings to strengthen the real-world applicability of exergame-based interventions for healthy aging.

## Data Availability

The raw data supporting the conclusions of this article will be made available by the authors, without undue reservation.
