# Supplementary material for: E-ACTIVE AGING study protocol: Evaluating an exergame-based and multicomponent exercise program for community-dwelling older adults at risk of falling
Source: Front Physiol. 2025 Dec 3;16:1691454. doi: 10.3389/fphys.2025.1691454 (PMC12708238; doi:10.3389/fphys.2025.1691454)
Supplement: Supplementary file 2 [file Table2.docx]

| **SET 1** | | | | | | | | | | **Month 1** |
| --- | --- | --- | --- | --- | --- | --- | --- | --- | --- | --- |
| Dorsal rotation | Rotation with inclination | Knee raises | Squats | Equilibrism | Dorsal rotation | Rotation with inclination | Knee raises | Squats | Running path |  |
|  |  |  |  |  |  |  |  |  |  |  |
| **SET 2** | | | | | | | | | |  |
| The warrior | The chair | Lunge with rotation | Squats | moto adductors | The warrior | Lunge with rotation | Knee raises | Squats | Monster's lair |  |
|  |  |  |  |  |  |  |  |  |  |  |
| **SET 3** | | | | | | | | | | **Month 2** |
| Crescent moon | Knee raises | Lateral inclination | Squats with extension | Trunk swinging | Crescent moon | Knee raises | Lateral inclination | Squats with extension | Jogging bridge |  |
|  |  |  |  |  |  |  |  |  |  |  |
| **SET 4** | | | | | | | | | |  |
| Lateral inclination | Side steps | The chair | Knee raises | Jump squats | Front inclination | Side steps | The chair | Knee raises | primaterra |  |
|  |  |  |  |  |  |  |  |  |  |  |
| **SET 5** | | | | | | | | | | **Month 3** |
| Lunge with rotation | Squats | The warrior (b) | Knee raises | Lateral inclination | Lunge with rotation | Squats | The warrior (b) | Knee raises | Jogging bridge |  |
|  |  |  |  |  |  |  |  |  |  |  |
| **SET 6** | | | | | | | | | |  |
| Sumo squats | Rotation with inclination | Side steps | Crescent moon with twist | They count pottery | Sumo squats | Rotation with inclination | Side steps | Crescent moon with twist | gran vía esporta |  |

Supplementary 2a. Description of the exergame list.
