# Supplementary material for: E-ACTIVE AGING study protocol: Evaluating an exergame-based and multicomponent exercise program for community-dwelling older adults at risk of falling
Source: Front Physiol. 2025 Dec 3;16:1691454. doi: 10.3389/fphys.2025.1691454 (PMC12708238; doi:10.3389/fphys.2025.1691454)
Supplement: Supplementary file 3 [file DataSheet1.pdf]

TIDieR

E-ACTIVE AGING study: Exergames-based training with a multicomponent exercise program for community-dwelling older adults at risk of falling.

**Details:**	multicomponent exercise program
**Why:**	Exergames-based training with multicomponent exercise may impact the strategies used to reduce the risk of falls and improve the quality of life of the elderly person.
**What (material):**	The intervention utilizes physical materials including dumbbells, color-coded resistance bands (Thera-Band®), chairs, and a Nintendo Switch® console with the *Ring Fit Adventure* exergame. Sessions are conducted using a 55-inch TV for visual feedback. The intervention is delivered by trained physiotherapists, who follow a structured progression protocol based on heart rate reserve and perceived exertion. Exercise sets and activities are tailored weekly and monthly.
**What (procedures):**	The intervention consists of 12 weeks of supervised exercise sessions, held twice weekly at a senior center. Each session includes a warm-up (5–10 minutes), a main activity (50 minutes), and a cool-down (5–10 minutes). Activities include strength, aerobic, balance, and flexibility exercises using dumbbells, resistance bands, and chairs. In the exergame group, participants additionally perform 20 minutes of interactive gameplay with *Ring Fit Adventure* on Nintendo Switch®, following a structured progression protocol. Physiotherapists monitor intensity using the Borg scale and vital signs. Pre-intervention sessions are conducted to train participants in equipment use and safety. All procedures are guided and supervised by physiotherapists trained in the intervention protocol, ensuring standardized delivery and participant safety.
**Who provided:**	The intervention is delivered by physiotherapists trained in the use of active exergames and multicomponent exercise protocols. Each physiotherapist was responsible for one intervention group and followed a structured, pre-defined exercise progression. They received specific training on monitoring exercise intensity using the modified Borg scale and vital signs, and on instructing participants in the safe and effective use of the Nintendo Switch® Ring Fit Adventure game. Their role included supervising each session, adapting exercises to participant needs, and ensuring adherence to the protocol.
**How (mode of delivery; individual or group):**	The intervention was delivered face-to-face at the SENAMA day center in Santiago, Chile. The multicomponent exercise group participated in group sessions, while the exergame-based training group performed the exergame portion in pairs and the multicomponent exercises in a group format. All sessions were supervised onsite by physiotherapists to ensure proper execution, safety, and adherence to the protocol.
**Where:**	The intervention took place at the SENAMA day center in Puente Alto, Santiago, Chile. This center provides social and health services to older adults and is equipped with the necessary infrastructure for physical activity sessions, including a dedicated exercise space, a 50-inch smart TV, and exercise equipment such as resistance bands, dumbbells, and chairs. The facility allows for both group and individual assessments, and ensures appropriate conditions of privacy, temperature, and humidity for conducting safe and effective interventions.

When and how much: The intervention was delivered over a 12-week period, with two sessions per week, totaling 24 sessions per participant. Each session lasted approximately 1 hour, consisting of a 5–10 minute warm-up, 50 minutes of main exercise activity, and a 5–10 minute cool-down. Exercise intensity was monitored using the modified Borg scale and heart rate reserve (HRR), with intensity progressively increasing throughout the intervention. Both groups followed structured protocols, with the exergame group completing 30 minutes of multicomponent exercise plus 20 minutes of exergame training per session.

Tailoring: The intervention was planned to be personalized and adapted according to each participant’s functional capacity and clinical characteristics. Exercise intensity was titrated individually using the modified Borg scale and heart rate reserve (HRR). The exergame sets were also progressively adjusted throughout the 12 weeks to match participants' physical capabilities and ensure appropriate challenge levels. Physiotherapists provided individualized instructions and adjustments during each session to maintain safety and effectiveness.

How well (planned): To ensure fidelity, the intervention was delivered by trained physiotherapists following a standardized protocol for both the multicomponent and exergame-based sessions. Exercise intensity was monitored in each session using the modified Borg scale and vital sign measurements (heart rate, blood pressure, oxygen saturation), and session structure was strictly adhered to (warm-up, main activity, cool-down). Adherence was tracked by recording attendance and participant-reported effort levels. Participants were encouraged to maintain consistent physical activity and nutritional habits, and any deviations were reported. These measures were planned to ensure the intervention was delivered as intended across all sessions and participants.
