# Supplementary material for: E-ACTIVE AGING study protocol: Evaluating an exergame-based and multicomponent exercise program for community-dwelling older adults at risk of falling
Source: Front Physiol. 2025 Dec 3;16:1691454. doi: 10.3389/fphys.2025.1691454 (PMC12708238; doi:10.3389/fphys.2025.1691454)
Supplement: Supplementary file 4 [file Table3.docx]

| Nº | **NAME EXERCISE** | **DESCRIPTION** |
| --- | --- | --- |
| 1 | Dorsal rotation | Requires continuously rotating trunk from side to side while maintaining an upright posture, with feet shoulder-width apart and holding the ring at waist level. |
| 2 | Rotation with inclination | From a semi-squat position, maintaining a slight forward flexion and holding the ring in front, alternating trunk rotations are performed, incorporating a pause under isometric tension at the point of maximum rotation. |
| 3 | Knee raises | Consists of alternately lifting the knees toward the chest in a continuous, coordinated movement with flexion and extension of the upper limbs. |
| 4 | Squats | With feet shoulder-width apart and holding the ring at abdomen level, controlled squats are performed, incorporating an isometric pause in semiflexion between the descent and ascent phases. |
| 5 | Lateral inclination | With feet shoulder width apart and holding the ring with raised arms, controlled lateral trunk inclinations are performed, incorporating an isometric pause at the point of maximum inclination. |
| 6 | Equilibrism | The objective is to capture as many coins as possible, which appear randomly while the avatar walks in tandem on a rail, carrying a horizontal bar and dodging obstacles and enemies. The participant must perform static walking while holding the ring at the level of the abdomen and perform rapid lateral trunk inclinations. |
| 7 | The warrior | Yoga posture involving lateral trunk bends with the lower limbs aligned in tandem, holding the ring with arms raised. |
| 8 | The chair | Yoga posture in which, starting from a static semi-squat, the upper limbs are moved in a controlled manner through flexion and extension. |
| 9 | Lunge with rotation | It requires stepping forward into a lunge position. While holding the ring with arms raised, trunk is rotated towards the leading leg, incorporating an isometric pause at the point of maximum rotation. |
| 10 | Moto adductors | The objective is to capture as many coins as possible, which appear randomly while the avatar advances in a cart, dodging obstacles and enemies. The participant sits with the ring between the knees exerting a variable force on it to control the cart's progress. |
| 11 | Crescent moon | Yoga posture in which alternate trunk rotations are performed with the lower limbs aligned in tandem and holding the ring in front. |
| 12 | Squats with extension | With feet externally rotated and placed wider than shoulder-width apart, holding the ring with arms raised, controlled squats are performed, incorporating an isometric pause in semi-flexion between the descending and ascending phases. |
| 13 | Trunk swinging | The objective is to capture as many coins as possible, which appear randomly while the avatar remains on a platform, dodging obstacles and enemies. The participant must hold the ring with arms raised and perform quick, coordinated trunk movements. |
| 14 | Lateral inclination | With feet shoulder width apart and holding the ring with raised arms, controlled lateral trunk inclinations are performed, incorporating an isometric pause at the point of maximum inclination. |
| 15 | Front inclination | Trunk is lowered forward with control from the hips, keeping the back straight. It mainly engages the abdominal muscles and the lower back. |
| 16 | Side steps | Sideways movement: take a wide step to one side, then return to the center and repeat on the other side. In addition to cardio, this strengthens the glutes and thighs, especially the abductor and adductor muscles. |
| 17 | Jump squats | A squat combined with an upward thrust. From the squat position, you rise explosively by extending your legs and arms (holding the Ring-Con), activating your glutes, quadriceps, calves, and arms. |
| 18 | The warrior (b) | Stand with your legs apart, one forward and one back in a semi-squat position, with your arms raised parallel to the floor holding the Ring-Con. Strengthens your legs and improves core stability. |
| 19 | Sumo squat | A variation of the squat with your legs wider apart and your toes pointing outward. As you lower yourself, focus on working your inner glutes, adductors, and quadriceps. Ideal for increasing hip mobility. |
| 20 | Crescent moon with twist | Exercise that combines hip opening and trunk rotation. Start with your front knee bent (in a crescent moon position), then rotate your trunk toward the side of your front leg while holding the Ring-Con. Works your glutes, oblique abdominals, and improves flexibility. |
| 21 | They count pottery | Fun exercise and stretching: simulate molding clay around the Ring-Con, making circular and pressing movements. This helps tone your arms and shoulders and improve the mobility of your hands and wrists. |
| 22 | Running path, Monster's lair, Jogging bridge, gran vía de esporta, primaterra | These running games aim to advance through various environments with obstacles and challenges. These levels demand adjusting pace, dodging barriers, facing challenges through continuous movement, and promoting aerobic exercise while progressing. Participants perform static marching or jogging while controlling the character's actions with the ring. |

Supplementary 2b. Description of the AVGs used in the protocol.
